# Randomized clinical trial of encapsulated and hand-mixed glass-ionomer ART restorations: one-year follow-up

**DOI:** 10.1590/1678-7757-2017-0129

**Published:** 2018-01-16

**Authors:** Maria Cristina Carvalho de Almendra Freitas, Ticiane Cestari Fagundes, Karin Cristina da Silva Modena, Guilherme Saintive Cardia, Maria Fidela de Lima Navarro

**Affiliations:** 1 Grupo Educacional DeVry, Faculdade DeVry FACID, Teresina, Piauí, Brasil; 2 UNESP – Univ. Estadual Paulista, Faculdade de Odontologia de Araçatuba, Departamento de Odontologia Restauradora, Araçatuba, São Paulo, Brasil; 3 Universidade do Sagrado Coração, Centro de Ciências da Saúde, Curso de Odontologia, Bauru, São Paulo, Brasil; 4 UniCesumar, Maringá, Paraná, Brasil; 5 Universidade de São Paulo, Faculdade de Odontologia de Bauru, Departamento de Dentística, Endodontia e Materiais Odontológicos, Bauru, São Paulo, Brasil

**Keywords:** Dental caries, Clinical trial, Glass ionomer cements

## Abstract

**Objective:**

This prospective, randomized, split-mouth clinical trial evaluated the clinical performance of conventional glass ionomer cement (GIC; Riva Self-Cure, SDI), supplied in capsules or in powder/liquid kits and placed in Class I cavities in permanent molars by the Atraumatic Restorative Treatment (ART) approach.

**Material and Methods:**

A total of 80 restorations were randomly placed in 40 patients aged 11-15 years. Each patient received one restoration with each type of GIC. The restorations were evaluated after periods of 15 days (baseline), 6 months, and 1 year, according to ART criteria. Wilcoxon matched pairs, multivariate logistic regression, and Gehan-Wilcoxon tests were used for statistical analysis.

**Results:**

Patients were evaluated after 15 days (n=40), 6 months (n=34), and 1 year (n=29). Encapsulated GICs showed significantly superior clinical performance compared with hand-mixed GICs at baseline (p=0.017), 6 months (p=0.001), and 1 year (p=0.026). For hand-mixed GIC, a statistically significant difference was only observed over the period of baseline to 1 year (p=0.001). Encapsulated GIC presented statistically significant differences for the following periods: 6 months to 1 year (p=0.028) and baseline to 1 year (p=0.002). Encapsulated GIC presented superior cumulative survival rate than hand-mixed GIC over one year. Importantly, both GICs exhibited decreased survival over time.

**Conclusions:**

Encapsulated GIC promoted better ART performance, with an annual failure rate of 24%; in contrast, hand-mixed GIC demonstrated a failure rate of 42%.

## Introduction

The Atraumatic Restorative Treatment (ART) approach is based on the removal of infected tooth tissues with hand instruments, followed by restoration of the cavity and sealing of adjacent pits and fissures^2^. This approach, which is an economical and effective method to prevent and control carious lesion development, causes less discomfort and dental anxiety to patients than the conventional rotatory instruments^2^.

Glass ionomer cements (GICs) have become the most used material for the ART approach due to their biological, physical, and chemical properties^17^. Notably, hand mixing of GICs might allow for an increased incidence of operator errors during material preparation, as the ratio of powder to liquid may vary according to manufacturer's recommendations^4^. The quantity of powder dispensed varies according to powder packing density in the volumetric scoop. The volume of liquid dispensed from the manufacturer-supplied dropper bottle varies depending on the angle at which the bottle is held, the pressure applied to squeeze a drop, and the inclusion of air bubbles^4^. With the purpose of decreasing these variables, encapsulated dental cements have been introduced in the market^21^. These premade mixtures utilize mechanical mixing methods and allow standardization of the powder/liquid ratio in a sealed capsule, which is expected to reduce variation in clinical outcomes^21^ ^,^ ^22^.

A meta-analysis of ART showed that high-viscosity GICs presented higher clinical survival rates than conventional or medium-viscosity glass ionomers^28^. This classification was only based on the powder/liquid ratio. However, a characterization of high viscosity GICs also considered improvement in the liquid components as well as changes in the powder^13^.

Some products are classified as medium-viscosity glass ionomers but are indicated by the manufacturers for ART techniques, and are available for hand mix or in capsules. Laboratory studies have shown that encapsulated GICs produce specimens with less porosity and higher mechanical strength than hand mix specimens^19^ ^,^ ^21^ ^,^ ^22^. However, there is no literature describing the survival rates of encapsulated versus hand-mixed GICs.

The purpose of this study was to evaluate the clinical performance of one conventional GIC (Riva Self-Cure, SDI Limited, Bayswater, VIC, Australia) supplied as both hand-mixed kits and in an encapsulated form. The null hypotheses to be tested were: 1) there is no difference in the survival rates of Class I restorations performed with hand-mixed or encapsulated GICs; and 2) there is no difference in the survival rates of GICs evaluated at different time periods.

## Material and methods

**Figure 1:**
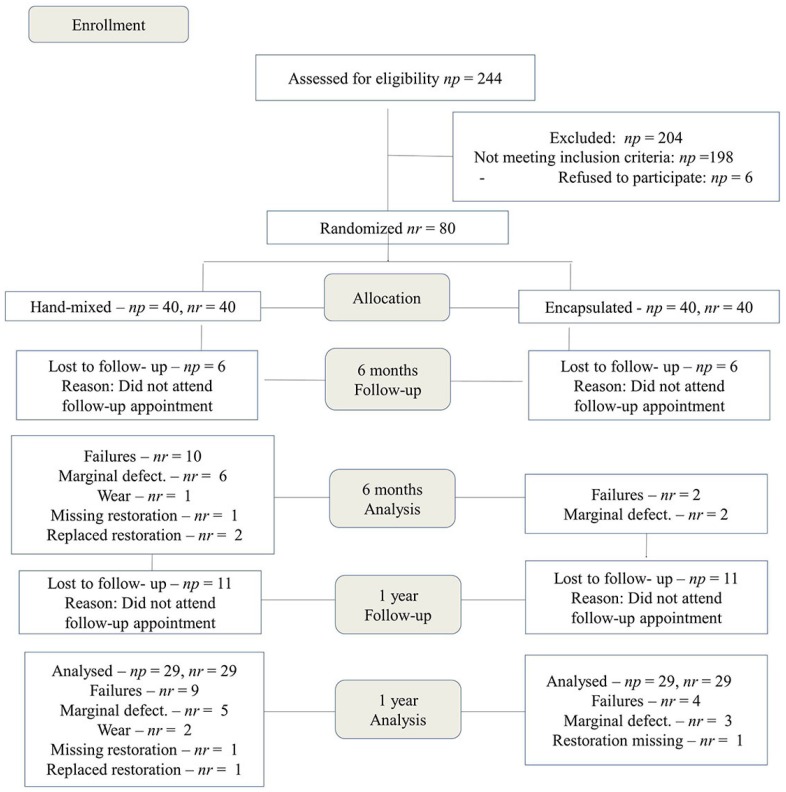
CONSORT participant flowchart. np=number of patients; nr=number of restorations

We performed a randomized and split-mouth clinical trial. Experimental design followed the Consolidated Standards of Reporting Trials (CONSORT) guidelines; the experimental flow chart is shown in Figure 1. Our local ethics committee approved the study (#095/2007) and it was also registered on REBEC (Brazilian Registry of Clinical Trials). The UTN (Universal Trial Number) of this study is U1111-1180-5126.

The study included 40 children from three public schools of suburban areas of the city of Bauru (northwest region of the state of São Paulo, Brazil) who presented at least two occlusal Class I carious lesions that involved dentin in permanent molars. Two carious lesions *per* child were randomly selected for restoration with hand-mixed or encapsulated forms of the conventional GIC (Riva Self-Cure, SDI Limited, Bayswater, VIC, Australia). Exclusion criteria included the presence of teeth with pulp exposure, a history of pain, or the presence of swelling or fistula. A total of 80 restorations were placed in children aged 11-15 years (mean: 12.98 ± 1.2 years). The patient cohort included 18 male and 22 female children.

We obtained informed consent forms from the legal guardians of all children recruited to the study. Then, we reviewed each child's record for demographic information, as well as their medical and dental history. Parents were asked to provide information about their socio-economic status, according to criteria from the Brazilian Association of Market Survey Institutes^3^.

Visible plaque index (VPI), gingival bleeding index (GBI), and decayed, missing and filled teeth (DMFT) index were assessed at baseline and recall appointments. The cold pulp test was used to determine pulp condition. Radiographs were taken to confirm clinical assessments. All children received oral health instruction. All clinical procedures were performed by one operator and one chairside assistant, who were both PhD students previously trained and calibrated on the ART approach. The restorations were performed at the Graduate Clinic of the Dental School.

The selected tooth was isolated with cotton rolls. Then, the chair assistant used a lottery method to randomly allocate the material (encapsulated or hand-mixed) used for each patient's first procedure. Initially, the tooth surface was cleaned with a wet cotton pellet. The ART approach was used to remove infected dentine with an excavator. Thin, unsupported enamel was carefully removed using a hatchet placed on the enamel with slight pressure. Local anesthesia was used, if necessary. Fissures adjacent to the cavity were gently cleaned with a probe. The clinical characteristics of all carious lesions were recorded by the operators. Distinction between active and inactive caries lesions was made on the basis of a combination of visual and tactile criteria: enamel/dentin cavity easily visible with the naked eye – surface of cavity feels soft or leathery on gentle probing in active lesions; enamel/dentin cavity easily visible with the naked eye – surface of cavity may be shiny and feels hard on probing with gentle pressure in inactive lesions^23^. The prepared cavity was then washed with a small cotton pellet soaked in water. A thin layer of calcium hydroxide cement (Hydro-C, Dentsply, York, PA, USA) was applied to the deepest cavities. Conditioning of the cavity and adjacent fissures was performed using a cotton pellet saturated with the liquid supplied for mixing of the GIC (polyacrylic and tartaric acids) for 10 seconds. Conditioned surfaces were washed three times with wet cotton pellets and dried with dry cotton pellets.

GICs were prepared according to the manufacturer's instructions. Chemical compositions of both GICs are presented in Table 1. For hand-mixed GIC, the filling material was inserted into the cavity using the smooth side of an excavator or a flat spatula for resin composite. Slight vibrations were made with the spatula on one side of the cavity margins for better adaptability of the GIC into the cavity, until filling the whole cavity. For encapsulated GIC, the plunger was placed on a hard surface and a mechanical mixer (Ultramat 2, SDI Limited, Bayswater, VIC, Australia, 4600 rpm) was used to mix the capsules for 10 seconds. The capsule was then placed into the Riva applicator (SDI Limited, Bayswater, VIC, Australia) to insert the GIC into the cavity. All adjacent pits and fissures were also sealed to prevent further caries.

**Table 1:** Chemical composition of glass ionomer cements(GICs)

Material	Composition		Powder / Liquid Ratio (g/g)	Batch Number
	Powder:	Weight %	Hand-mixed:	Hand-mixed:
Riva Self Cure Capsules and hand-mixed versions	Fluoro Aluminosilicate glass Polyacrylic acid	90 to 95 5 to 10	3.1:1	P: 100607 L: 100715
	**Liquid:**		**Capsules:**	**Capsules:**
	Polyacrylic acid	20 to 30	3.2:1	50711EG
	Tartaric acid	10 to 15		
Riva Coat	Acrylic monomer			

After inserting the GIC, a gloved finger coated with petroleum jelly was used to apply pressure to the GIC for 1 minute. Occlusion was checked and excess material was removed with a carver. Restorations were coated with a layer of petroleum jelly to prevent sorption during occlusal checking. Subsequently, petroleum jelly was removed from the surface using at least two cotton wool pellets. Riva Coat (SDI Limited, Bayswater, VIC, Australia) was applied to the surfaces of final restorations and light-cured for 20 seconds (Astralis 10, 650 mW/cm^2^, Ivoclar Vivadent, Schaan, Liechtenstein). Both restorations were performed at the same appointment.

Upon completion of GIC application, the following information were recorded: whether anesthesia or pulp protection were required, and whether post-operative sensitivity was present.

**Figure 2:**
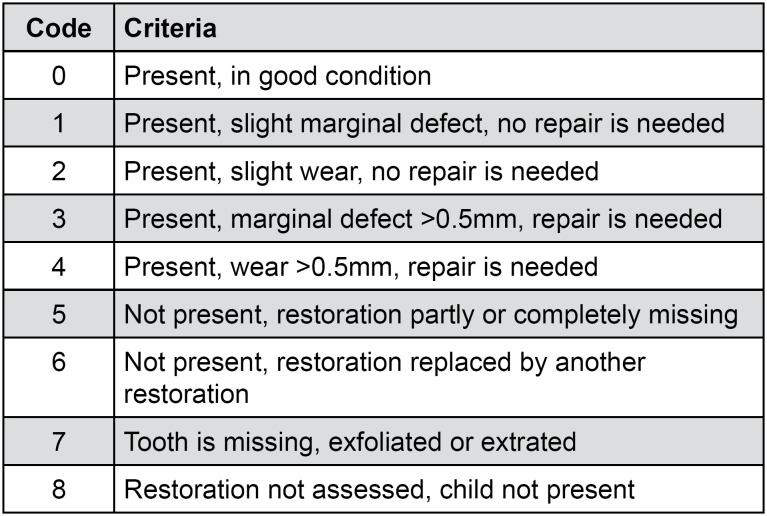
ART criteria according to Lo and Holmgren^12^ (2001) Codes: 0, 1, 2 = successful; 3, 4, 5, 6 = failure; 7,8 = excluded

Restorations were evaluated after 15 days (baseline), 6 months, and 1 year, using the criteria established for ART restorations (Figure 2)^14^.

Sample size (n) was calculated, using a proportional comparison formula for two-tailed test. Significance sequence (Zα) and statistical power (Zβ) were adopted in 5% and 80%, respectively. The non-effectiveness ratio of encapsulated and hand-mixed GIC is respectively 5.3%^29^ and 28.3%^30^. To offset any losses during the study, 15% were added to the amount found. Therefore, the initial sample size was set at 40 restorations for each group (http://www.lee.dante.br).

Evaluations were performed by two calibrated independent examiners who did not include the operator, allowing a blinded study for both participants and evaluators. The examiners used World Health Organization CPI probes and plane front surface mirrors^14^. The evaluators examined each restoration consecutively and final assessment was made based on consensus. Inter-examiner agreement was calculated using the Kappa coefficient.

The Wilcoxon matched pairs test was used to compare: the level of anxiety of patients before and after the treatment; the characteristics of the patients’ oral health at different periods; the distribution of lesion characteristics and perception of clinical procedures between both groups; and the survival of encapsulated and hand-mixed GICs at each evaluation period. Additional intragroup comparisons were performed between baseline and other evaluation periods. A multivariate logistic regression was performed regarding GIC presentation, type of teeth involved, activity of the lesion, postoperative sensitivity, and pulpal protection. The Gehan-Wilcoxon test was used to analyze survival of the restorations as a function of the two forms of GICs. Statistica v. 12 (StatSoft Inc., Tulsa, OK, USA) was used and the level of significance was set at *p*=0.05.

## Results

The socio-economic status assessment indicated that 77.5% of the participants were classified as class C; 20% as class D; and 2.5% as class E. No perception of patient pain or discomfort was observed in 52.5% of teeth; minor pain was observed in 33.8% of teeth; and severe pain was observed in 13.7% of teeth. Application of anesthesia was performed in 12.5% of teeth.

Statistically significant reduction in the VPI index between baseline and one year was observed (*p* = 0.007). We did not find any statistically significant differences in the GBI index (*p* ≥ 0.05). We found statistically significant differences in the DMFT index between baseline and 6 months (*p* = 0.017), and between baseline and 1 year (*p* = 0.010).

The distribution of lesions and clinical procedure characteristics between encapsulated and hand- mixed GICs is shown in Table 2. We did not find any statistically significant differences on the distribution of teeth, lesions, cavities, and restorations characteristics between the evaluated groups.

**Table 2:** Distribution of teeth, lesions, cavities, and restorations characteristics in percentage

Characteristics	Hand-mixed	Encapsulated
**Teeth**		
Mandibular first molar	40	37.5
Maxillary first molar	32.5	35
Mandibular second molar	20	22.5
Maxillary second molar	7.5	5
**Activity of lesion**		
Active	42.5	42.5
Inactive	57.5	57.5
**Protection with calcium hydroxide**		
Yes	12.5	15
No	87.5	85
**Postoperative sensitivity at baseline**		
Absent	95	82.5
Present, during one day	2.5	7.5
Present, during more than one day	2.5	5
Still present	0	5

Patients were evaluated after 6 months (n = 34; 85%) and 1 year (n = 29; 72.5%). The primary reason for patient drop-out was change of address: to other parts of the city, rural areas, or other cities. To reach patients during follow-up periods, we consulted patient chart information, as well as parents and friends’ addresses and phone numbers and public school records.

Inter-examiner kappa coefficient values were 0.89, 0.81, and 0.89 for baseline, 6-month and 1-year evaluations, respectively. Hand-mixed GIC restorations presented 15% failures (6 restorations) at baseline while encapsulated restorations did not present any failure in this period, only two slight defects. Encapsulated GICs showed significantly superior clinical performance compared with hand-mixed GICs at baseline (*p*=0.017), 6 months (*p*=0.001), and 1 year (*p*=0.026). For hand-mixed GICs, we observed statistically significant differences only between the period of baseline to 1 year (*p*=0.001). There was a statistically significant difference between the clinical performance for the following periods: 6 months to 1 year (*p*=0.028) and baseline to 1 year (*p*=0.002) for encapsulated GIC (Table 3).

**Table 3:** Distribution of Atraumatic Restorative Treatment (ART) scores, according to the evaluated groups at baseline, 6 months, and 1 year

	Baseline		6 months		1 year	
Scores	Hand	Capsule	Hand	Capsule	Hand	Capsule
	mixed		mixed		mixed	
0	29 (72.5%)	38 (95%)	14 (41.3%)	25 (73.5%)	7 (24.1%)	16 (55.2%)
1	2 (5%)	1 (2.5%)	3 (8.8%)	5 (14.7%)	5 (17.2%)	3 (10.3%)
2	3 (7.5%)	1 (2.5%)	7 (20.6%)	2 (5.9%)	8 (27.6%)	6 (20.7%)
3	4 (10%)	-	6 (17.6%)	2 (5.9%)	5 (17.2%)	3 (10.3%)
4	1 (2.5%)	-	1 (2.9%)	-	2 (6.9%)	-
5	1(2.5%)	-	1 (2.9%)	-	1 (3.5%)	1 (3.5%)
6	-	-	2 (5.9%)	-	1 (3.5%)	-
7	-	-	-	-	-	-
8	-	-	6	6	11	11
Total	sucess:85%	sucess:100%	sucess:70.7%	sucess:94.1%	sucess:68.9%	sucess:86.2%
	failure:15%	failure:0%	failure:29.3%	failure:5.9%	failure:31.1%	failure:13.8%

Logistic regression analysis showed that no variables studied had statistical influence on the clinical performance of GICs (Table 4). There were significant differences in the cumulative survival rates of encapsulated and hand mixed GICs over one year (*p*=0.005); however, both GICs showed decreased success over time.

**Table 4:** Multivariate logistic regression for different variables

Variables	OR	Estimate	95%CI		P-value
GIC	0.4298	0.7477	0.0992	18.610	0.259
Teeth	0.3545	0.9590	0.0541	23.228	0.280
Activity of lesion	0.3142	0.7871	0.0671	14.699	0.141
Protection with calcium hydroxide	0.3550	13.201	0.0267	47.207	0.433
Postoperative sensitivity at baseline	0.4749	12.343	0.0422	53.379	0.546

Odds Ratio (OR), Confidence Interval (CI)

## Discussion

In this study, the best clinical performance was achieved by performing restorations with encapsulated GICs. It has been suggested that encapsulated GICs might be a potential solution to the operator-induced variables observed with use of hand-mixed GICs^22^.

The study design chosen was a split-mouth randomized controlled trial, in which the two interventions were randomly allocated to different teeth in the same oral cavity. Relative to a parallel design, a split-mouth design has the advantage of removing most of the patient outcome variability from the intervention effect estimate to achieve a potential increase in statistical power^26^. Additionally, there were no significant differences between groups in the distribution of lesion characteristics and perception of clinical procedures. This uniform distribution of data supports the use of a randomized experimental design in a clinical study. The 6-month and 1-year drop-out rates were 15% and 27.5%, respectively, similar to the 1-year follow-up rate of a prior ART study (28.6%)^20^.

The population in our study included middle (class C) or lower (class D or E) social classes, using a socio-economic classification that divides the population into categories according to consumption potential and level of education of the head of the household^3^. Prior studies have shown that low income is related to high caries index in the early stages of life^11^.

The VPI score statistically decreased after one year, which is likely due to the hygiene instructions provided in all appointments. The DMFT was very high at all evaluation periods (4.8 at baseline, 5.2 at 6 months, 5.3 at 1 year) and showed a significant increase between baseline and 6 months. This finding seems contradictory with the VPI score, but it can be explained by the additional treatment provided to patients that increase the DMFT index, since proximal carious lesions not detected initially were treated.

An *in vitro* study showed more discrepancies at margins of GIC restorations lined with non-setting calcium hydroxide in comparison with GICs lined with setting calcium hydroxide. In this study, only 12.5 to 15% of the restorations were lined with setting calcium hydroxide and no statistical influence was observed on clinical performance of both hand-mixed and encapsulated GICs^24^. The amount of lining and its extension could interfere with the performance of the restorations^24^. In this study, the lining covered only a small area the operator suspected could be too closed to pulp.

It has been shown that encapsulated restorative GICs have significantly greater compressive strength, elastic modulus and *in vitro* wear-resistance when compared with their hand-mixed counterparts^7^. A recent study demonstrated that two encapsulated high-viscosity glass-ionomers (EQUIA system and Chemfil Rock) had significantly higher test values for diametral tensile, flexural, and compressive strengths than the commonly used hand-mixed high-viscosity glass-ionomers^19^.

The manipulation of GICs in lower powder-to-liquid ratios than those recommended by the manufacturer has been reported to significantly reduce mean compressive fracture strength^10^. Additionally, optimum posterior glass-ionomer restorative cement properties may be compromised by variations in temperature and relative humidity, often encountered in clinical practices in which the materials are hand-mixed^4^. The hand-mixed technique may result in an unbalanced distribution of unreacted glass filler particles in the plastic mass^10^. If insufficient pressure is applied during the manipulation process, these unreacted glass filler particles can form agglomerates that contain voids susceptible to cracking when the material is stressed under load^9^. A study on porosity showed that hand-mixed cements presented greater porosity than encapsulated cements^22^. When luting and restorative GICs were compared, the total volume ratio of bubbles was statistically different between hand-mixed and encapsulated GICs only for more fluid luting types of GICs^22^. The main factor that affects the success of ART restorations is the operator skills regarding the technique^15^. To avoid this variable, only one trained operator made all restorations.

A study carried out by Nomoto and McCabe^21^ (2001) observed that hand-mixed restorative GICs presented a significantly lower compressive strength than GICs mixed by rotation. Although the method of mixing can markedly influence material properties, the powder/liquid ratio and initial viscosity may also have some effects on the material properties, as shown by comparing the same generic materials from the same manufacturer^21^ ^,^ ^22^.

In a study in which physical properties of hand- mixed Riva Self Cure were studied, the 1-week compressive strength was maintained through 1 year. However, the 1-week surface hardness was only maintained through 6 months^25^. A progressive wear was also observed for hand-mixed Riva Self Cure through 1 year in a laboratory study^5^. When this GIC was compared to high-viscosity cements, it demonstrated similar compressive strength, but lower flexural strength and microhardness^5^.

The literature reports survival rates for single-surface ART restorations using high-viscosity glass-ionomers similar or superior to those achieved with amalgam restorations after 6 years^12^ ^,^ ^18^. Some studies found survival rates of 97.3% at 6 months and of 98.6% at 1-year follow-up^6^ ^,^ ^11^. Another study from Souza, et al.^27^ (2003) showed a success rate of 86.2% for occlusal restorations performed with Fuji IX and 88.4% for those restored with Fuji Plus at eight months post-treatment. Nevertheless, the large majority of ART restoration survival studies have used high- viscosity hand-mixed GICs. Therefore, it is difficult to discuss the effect of different modes of mixing GIC on the survival of single-surface ART restorations. In a meta-analysis of ART, the cumulative survival rates of single-surface ART restorations in permanent teeth over the first three years was 85% (CI, 77-91%)^1^. In this study, the cumulative survival rates were lower for the hand-mixed form – 58.1% (CI, 40.1-76.1) – when compared to encapsulated GIC – 75.7% (CI, 56.1-95.3) – over a 1 year evaluation period. It is important to emphasize that, according to a metaanalysis, the powder/liquid ratio used for Riva (3.1:1) is considered as medium-viscosity glass-ionomers (1.5:1≥ powder:liquid ≤3.6:1)^28^. This fact may have influenced the low survival rate encountered in this study. However, the directions of the manufacturer indicate it for ART technique.

Main problems with prospective studies are recall rate, adequate sample size, and control of the baseline conditions. Only twenty-nine patients out of the original sample were included in the final analysis been a limitation of this study. This recall number is more than the recall rate of 66.2% from a study by Mickenautsch, et al.^16^ (2000), but less compared to the study by Farag, et al.^8^ (2011). However, the sample size of this study was large enough for statistical significance.

## Conclusion

Based on our present results, encapsulated GICs appear to promote better ART performance, contrasting an annual failure rate of 24% with 42% for hand-mixed GICs. Encapsulated GICs may be a more promising option for the ART approach than their hand-mixed equivalents.
